# Hepatitis C care cascade in a large academic healthcare system, 2012 to 2018

**DOI:** 10.1097/MD.0000000000032859

**Published:** 2023-03-10

**Authors:** Jasmine Nakayama, Vicki S. Hertzberg, Joyce C. Ho, Roy L. Simpson, Emily J. Cartwright

**Affiliations:** a Emory University Nell Hodgson Woodruff School of Nursing, Atlanta, GA; b Emory University Department of Computer Science, Atlanta, GA; c Emory University School of Medicine, Atlanta, GA; d Atlanta VA Medical Center, Atlanta, GA.

**Keywords:** chronic hepatitis C, care cascade, electronic medical records, viral hepatitis

## Abstract

To determine the hepatitis C virus (HCV) care cascade among persons who were born during 1945 to 1965 and received outpatient care on or after January 2014 at a large academic healthcare system. Deidentified electronic health record data in an existing research database were analyzed for this study. Laboratory test results for HCV antibody and HCV ribonucleic acid (RNA) indicated seropositivity and confirmatory testing. HCV genotyping was used as a proxy for linkage to care. A direct-acting antiviral (DAA) prescription indicated treatment initiation, an undetectable HCV RNA at least 20 weeks after initiation of antiviral treatment indicated a sustained virologic response. Of the 121,807 patients in the 1945 to 1965 birth cohort who received outpatient care between January 1, 2014 and June 30, 2017, 3399 (3%) patients were screened for HCV; 540 (16%) were seropositive. Among the seropositive, 442 (82%) had detectable HCV RNA, 68 (13%) had undetectable HCV RNA, and 30 (6%) lacked HCV RNA testing. Of the 442 viremic patients, 237 (54%) were linked to care, 65 (15%) initiated DAA treatment, and 32 (7%) achieved sustained virologic response. While only 3% were screened for HCV, the seroprevalence was high in the screened sample. Despite the established safety and efficacy of DAAs, only 15% initiated treatment during the study period. To achieve HCV elimination, improved HCV screening and linkage to HCV care and DAA treatment are needed.

## 1. Introduction

Affecting an estimated 2.8 million people in the United States,^[1]^ chronic hepatitis C virus (HCV) is a large public health problem that disproportionately affects “baby boomers,” (i.e., those born during 1945–1965).^[2]^ It is estimated that this birth cohort accounts for approximately 80% of people living with HCV in the United States.^[2,3]^ Because HCV can cause hepatic and extrahepatic damage for many decades after initial infection without any noticeable signs or symptoms, complications related to untreated HCV are peaking among baby boomers who were infected 30 or more years ago.^[4,5]^ Left untreated, HCV causes cirrhosis in approximately 20% to 25% of patients, increasing the risk for end-stage liver disease, hepatocellular carcinoma, and death.^[5]^ HCV is associated with higher liver related and all-cause mortality and is a leading cause of liver transplantations in the United States.^[6–8]^ Between 2005 to 2014, baby boomers had the highest rate of inpatient stays involving HCV compared with older and younger adults.^[9]^ In 2012, the centers for disease control and prevention recommended that everyone in the baby boomer birth cohort receive HCV screening.^[10]^ The United States preventive services task force recommended universal screening for those born during 1945 to 1965 in 2013 and then for all adults aged 18 to 79 years in 2020, giving the recommendation a B grade.^[11,12]^

The introduction of all oral, interferon-free, direct-acting antiviral (DAA) medications in 2013 dramatically changed treatment options for HCV by increasing access to safer and more effective therapies compared to previous interferon-based therapies.^[13–15]^ Treatment with DAA therapies leads to a sustained virologic response (SVR) in over 95% of persons treated.^[14,16]^ Achieving an SVR significantly reduces the incidence of hepatocellular carcinoma, liver-related mortality, and all-cause mortality.^[17–20]^ The arrival of DAA treatment, which produced high rates of SVR, led the world health organization to set a goal to eliminate HCV as a public health threat by 2030.^[21]^ Elimination will only be possible if persons with HCV are successfully identified, linked to care, and treated with DAA therapies.^[22,23]^

The “care cascade” model is useful for visualizing steps in the sequence of medical care from disease identification to treatment.^[24]^ The model was initially used to describe the various steps of care for human immunodeficiency virus: screening, linkage to care, initiation and continuation of antiretroviral medications, and viral suppression.^[25]^ Since then, the model has been adapted to describe steps of care for HCV in diverse settings and patient populations, such as those who are incarcerated, who inject drugs, or who are co-infected with human immunodeficiency virus.^[26–33]^ By measuring each step of care, the model monitors progress, reveals gaps in care, and identifies areas for improvement. While a few studies have focused on the HCV care cascade in the 1945 to 1965 birth cohort,^[34–37]^ none have exclusively examined this birth cohort in the Southern United States, a region with high numbers of persons with HCV.^[38]^ The purpose of this study is to describe the current HCV care cascade among the 1945 to 1965 birth cohort at a large academic healthcare system during the initial years when DAA medications became available, which may assist public health officials and clinicians in providing better care to persons with HCV and in working toward HCV elimination.

## 2. Methods

### 2.1. Data source and analytic strategy

Data were extracted from an existing electronic healthcare record (EHR) database housed at a research university’s school of nursing and developed in partnership with a large academic healthcare system in the Southern United States.^[39,40]^ The EHR database contains 811,771,561 unique electronic records from 1,089,586 unique patients, which is a 17% simple random sample of all patients seen in the healthcare system between 2012 and 2017 with no restrictions on demographics, diagnoses, or services received. The records were taken from the Clinical Data Warehouse and deidentified to remove all protected health information through shifting dates, truncating zip codes, anonymizing patients identification numbers, and removing patients names and contact information in compliance with safe harbor methods by the data solutions group.^[41]^ The Emory University Institutional Review Board provided a waiver for this study of deidentified data.

The database was accessed via the relational database management system PostgreSQL. EHR data regarding patients demographic information, healthcare services, laboratory results, medication histories were extracted from the database into comma-separated values files and analyzed using RStudio, and an integrated development environment for the R programming language.

### 2.2. Study sample

Adults in the 1945 to 1965 birth cohort who received outpatient services formed an analytic subset, and it was then determined who were screened for HCV. The sample was limited to those who received outpatient care in the healthcare system on or after January 1, 2014. Data were available for HCV care received through 2018. This specification excluded patients who received acute care services in the healthcare system but may have received outpatient care elsewhere.

### 2.3. Care cascade measures

The number of patients in each step of the care cascade was determined: screening and confirmatory testing, linkage to care, initiation of DAA, SVR. Laboratory results for HCV antibody and HCV viral tests (i.e., ribonucleic acid [RNA] and genotype) were used to determine screening and confirmatory testing, and respectively. This healthcare system has employed reflex testing for HCV RNA since 2012. Although the clinical test for HCV screening is the HCV antibody, this study assumed that anyone with test results for HCV RNA or genotype was previously screened with an HCV antibody test even if 1 was not recorded, accounting for patients who may have had HCV antibody testing before 2014 and then had confirmatory testing during the study period. This criterion also accounted for patients who received HCV screening at outside facilities and entered the healthcare system for follow-up care.

**Figure 1.**
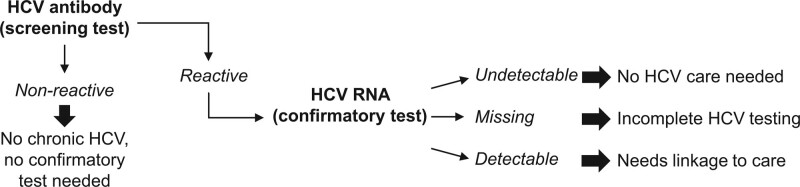
Flowchart in determining patients statuses in chronic hepatitis C screening and confirmatory testing.

It was determined which screened patients did not have chronic HCV, were lost to follow-up, or needed linkage to care according to results of HCV antibody and viral tests (Fig. 1). HCV RNA test results of < 15 International Units per liter were considered undetectable. Seronegative patients, those with nonreactive HCV antibody tests, did not have HCV and did not need confirmatory testing or HCV care. Seropositive patients had reactive HCV antibody tests. Seropositive patients with undetectable HCV RNA did not need further HCV care, and seropositive patients with missing HCV RNA testing were considered to have incomplete HCV testing. Seropositive patients with detectable HCV RNA had chronic HCV and needed HCV care. If a patient had a recorded HCV genotype but no recorded RNA, it was assumed that the patient had detectable RNA and thus needed HCV care. This study used the HCV genotype test as a proxy for determining linkage to care (i.e., meeting with a medical professional who provides HCV treatment and management). However, if a patient had a record of initiating antiviral treatment without a recorded genotype, it was assumed that the patient was linked to care.

Initiation of antiviral treatment was defined as the presence of any of the following in the patient’s medication history: boceprevir, daclatasvir, dasabuvir, elbasvir, glecaprevir, grazoprevir, interferon alfacon-1, interferon alpha-2a, interferon alpha-2b, ledipasvir, ombitasvir, paritaprevir, pegylated interferon, pegylated interferon alpha-2b, pibrentasvir, ribavirin, ritonavir, simeprevir, sofosbuvir, telaprevir, velpatasvir, and voxilaprevir. Date of initiation of antiviral treatment was considered the first date that any of the medications were recorded. While treatment durations can vary from 8 to 24 weeks, this study defined SVR as the presence of undetectable HCV RNA at least 20 weeks after initiation of antiviral treatment and on any subsequent test.

Differences in the number of patients between steps indicated drop-offs between steps. For instance, patients who had been linked to care but did not initiate antiviral treatment were considered to have dropped off in the care cascade after the linkage to care step. Additionally, recorded deaths in the EHR were considered in determining progress through the care cascade.

## 3. Results

**Figure 2.**
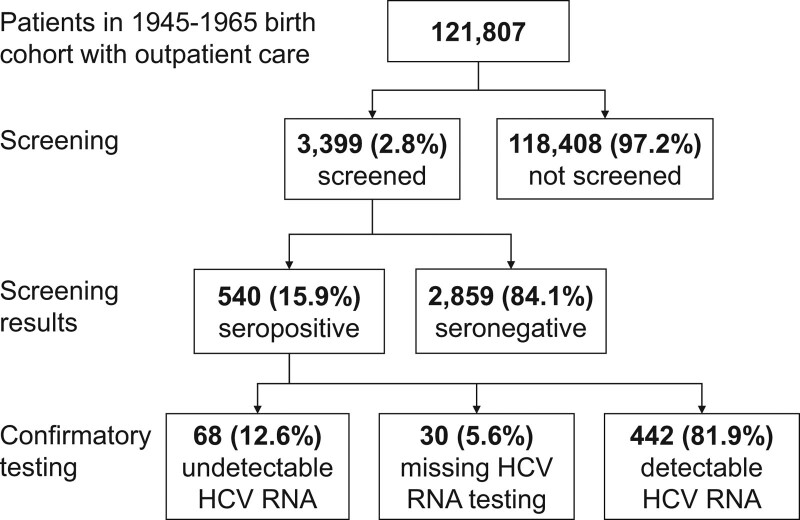
Chronic hepatitis C screening and confirmatory testing results among 1945 to 1965 birth cohort at large academic healthcare system. Footnote: Percentages do not add up to 100% due to rounding.

Of the 121,807 patients in the 1945 to 1965 birth cohort who received outpatient care on or after January 1, 2014, 3399 (2.8%) patients were screened for HCV between January 1, 2014 and June 1, 2017 (Fig. 2). Of the 3399 patients, 2859 (84.1%) were seronegative and did not need confirmatory testing or HCV care; the remaining 540 (15.9%) were HCV seropositive. Of the 540 patients, 68 (12.6%) had an undetectable HCV RNA, 30 (5.6%) patients had missing HCV RNA testing, and 442 (81.9%) patients had detectable HCV RNA (percentages do not add up to 100% due to rounding).

**Figure 3.**
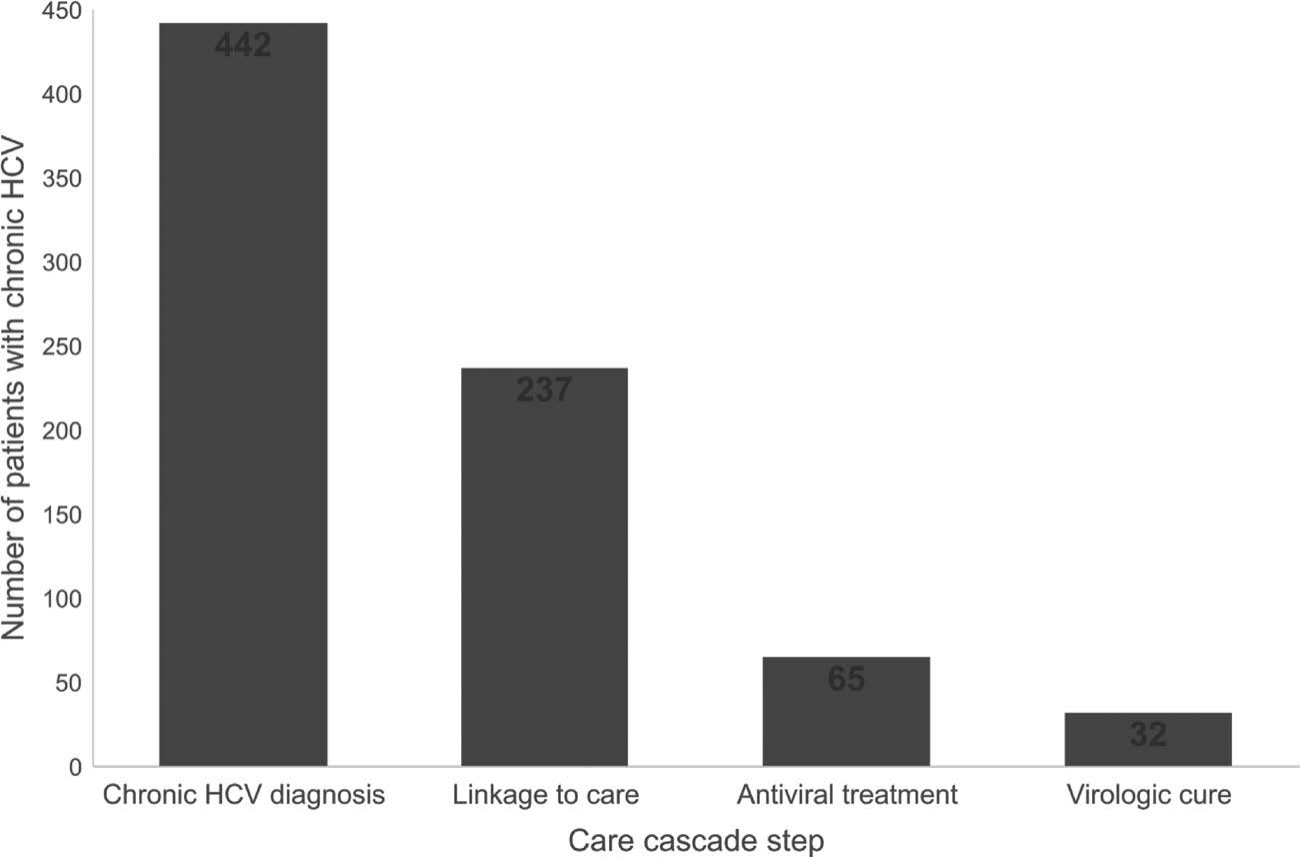
Chronic hepatitis C care cascade among 1945 to 1965 birth cohort patients at large academic healthcare system.

Figure 3 presents the care cascade for the 442 patients with chronic HCV. Among those patients, 237 (53.6%) were linked to care and 18 (4.1%) died prior to linkage to care. DAA treatment was initiated in 65 patients (27.4% of those linked to care and 14.7% of all patients with chronic HCV). Seven deaths occurred after linkage to care and before initiating DAA treatment. The following medications were identified: daclatasvir, dasabuvir, elbasvir, grazoprevir, ledipasvir, ombitasvir, paritaprevir, ribavirin, ritonavir, simeprevir, sofosbuvir, velpatasvir, and voxilaprevir. Three deaths occurred in the group that initiated DAA treatment. Finally, 32 (49.2% of those who initiated antiviral treatment), achieved SVR. Thus, 7.2% of patients with chronic HCV completed the care cascade.

## 4. Discussion

This study identified low HCV screening, linkage to care, and treatment in this EHR analysis of patients in a 1945 to 1965 birth cohort who received outpatient services in a large academic healthcare system in the Southern United States. Based on prior national surveys, an estimated 3.2% of persons born during 1945 to 1965 in the United States are HCV seropositive.^[42]^ However, we found that 16% of those tested for HCV were seropositive in our study. Only 54% of patients with chronic HCV were linked to care, and of those linked, only 27% initiated DAA treatment during the study period. There was no established program during the study period to address HCV screening, linkage, or treatment; this analysis represents the baseline or “usual state” of HCV care at the healthcare system. Using the EHR to create an HCV care cascade identified patients with untreated chronic HCV and will allow for future, in-depth exploration of the reasons for drop-off at each step.

A low percentage (3%) of the baby boomer birth cohort had been screened for HCV during the study period despite centers for disease control and prevention and United States preventive services task force recommendations.^[10,11]^ While it is possible that HCV screening occurred before or after the study period, other studies have identified similarly low rates, especially in commercially insured populations. An analysis of data from the 2015 National Health Interview Survey indicated that 13.8% of baby boomers reported ever undergoing HCV screening, and the percentage was lower in those with private insurance (12.8%) compared with public insurance (Medicaid and/or Medicare, 26.1%).^[43]^ HCV screening rates in correctional settings, Veterans Affairs, and safety net hospitals are substantially higher with reported rates ranging from 60% to 90%.^[44–46]^ Clinical decision support prompts and opt-out options have been shown to increase HCV screening.^[35]^

Approximately half of patients with chronic HCV were linked to care, a critical step in the pathway to curative DAA treatment. The large drop-off between HCV testing and linkage to care reveals gaps in connecting patients with HCV to clinicians trained and equipped to provide DAA treatment. Other studies have indicated that a dedicated navigator can increase the proportion that are linked to HCV care up to 85%.^[47]^ Without an intensified navigation program, other studies report 56% to 65% achieve HCV care linkage.^[37,48]^ Additional strategies to decrease this drop-off include training more clinicians to treat HCV, improving care coordination, integrating HCV care into current healthcare facilities, and providing HCV care at nontraditional facilities, including the use of telehealth.^[49–51]^

This study indicated that most of the patients who were linked to care in our analysis did not initiate antiviral treatment. The arrival of safe and highly effective DAA treatment should substantially reduce the drop-off between linkage and treatment initiation; in fact, recently published HCV care cascades no longer report linkage and instead focus on DAA treatment receipt as the next step after HCV diagnosis.^[52]^ Unfortunately, significant barriers to curative DAA therapies exist, including racial discrimination, limited healthcare access, underinsurance, or lack of insurance.^[53–55]^ Opportunities to improve this gap include addressing systemic and clinician-level biases, partnering with insurers to support treatment, supporting patients in navigating medication approval processes, and providing social services to tackle patient barriers.^[51,56,57]^ Even insured patients may experience difficulties accessing DAA treatment related to payor restrictions and denials; described interventions include appealing preauthorization denials, waiting for specific DAAs to appear on approved formularies, and dedicating staff to assist with navigating the DAA treatment approval process for patients.^[55,58,59]^

This study is subject to several limitations. Due to date shifting to deidentify the dataset, there may be misclassification of patients in the 1945 to 1965 birth cohort. Additionally, it is possible that patients progress through the care cascade was misclassified due to unavailability of data, such as record of HCV care received in another facility outside of the healthcare system or specialist referral. This study did not have access to data regarding patients disease severity, and its definition of SVR may have overcounted patients who required longer treatment durations. Patients screened for HCV prior to 2012 without subsequent testing were not included in analysis. Reasons for dropping out of the care cascade and data on patients’ relocation, reinfection, or relapse were not described in this study. It was indeterminate whether some patients who dropped off after testing or linkage to care experienced spontaneous clearance. Furthermore, there may be facility-specific or clinician-specific variations in HCV care within this healthcare system that were not captured in these data. Differences in defining certain steps can limit comparison with descriptions of cascades of care from other studies.

This study identified that only 7% of baby boomers with HCV completed the care cascade at a large academic healthcare system, highlighting systematic and individual opportunities for improvement at every step of the HCV. Suggested strategies to decrease these gaps include training more clinicians in HCV management, improving care management and coordination, and increasing access to DAAs. The results from this study guided quality improvement efforts at this academic healthcare system to identify all patients with untreated chronic HCV and link them to care. Using EHR data to create an HCV care cascade can assist healthcare systems in identifying drop-offs in HCV screening, linkage, and treatment. More resources devoted to this public health problem can bring the nation closer to the worldwide goal of HCV elimination by 2030.

## Author contributions

**Conceptualization:** Jasmine Nakayama, Vicki S. Hertzberg, Emily J. Cartwright.

**Data curation:** Jasmine Nakayama, Vicki S. Hertzberg, Roy L. Simpson.

**Formal analysis:** Jasmine Nakayama.

**Investigation:** Jasmine Nakayama.

**Methodology:** Jasmine Nakayama, Emily J. Cartwright.

**Resources:** Jasmine Nakayama, Vicki S. Hertzberg.

**Software:** Jasmine Nakayama.

**Supervision:** Vicki S. Hertzberg, Joyce C. Ho, Roy L. Simpson, Emily J. Cartwright.

**Visualization:** Jasmine Nakayama.

**Writing – original draft:** Jasmine Nakayama.

**Writing – review & editing:** Jasmine Nakayama, Vicki S. Hertzberg, Joyce C. Ho, Roy L. Simpson, Emily J. Cartwright.
